# Synergistic Anti-Tumor Activity of LRPPRC Inhibition and Dasatinib Through Dual Oxidative Phosphorylation Disruption

**DOI:** 10.3390/ph19030472

**Published:** 2026-03-12

**Authors:** Jing Chen, Lu Gao, Yuxin Liang, Wei Zhou, Yong Wang, Xiaojia Wang, Xiaohong Fang, Xiying Shao

**Affiliations:** 1 The Second Clinical Medical College, Zhejiang Chinese Medical University, Hangzhou 310053, China; 2 Hangzhou Institute of Medicine (HIM), Chinese Academy of Sciences, Hangzhou 310018, China; 3 Zhejiang Cancer Hospital, Hangzhou Institute of Medicine (HIM), Chinese Academy of Sciences, Hangzhou 310022, China

**Keywords:** LRPPRC, Dasatinib, synergistic inhibition, high-throughput screening, combination therapy

## Abstract

**Background/Objectives**: Mitochondrial Oxidative Phosphorylation (OXPHOS) is a critical metabolic dependency in many cancers. Targeting OXPHOS through Leucine-Rich PPR Motif-Containing Protein (LRPPRC) degrader-mediated OXPHOS Complex Biogenesis Inhibition (OCBI) has demonstrated promising anti-tumor activity. However, rational combination strategies to enhance therapeutic efficacy remain undefined. This study aims to identify FDA-approved drugs that synergize with LRPPRC inhibition and elucidate the underlying mechanism. **Methods**: We conducted a high-throughput screen of 1376 FDA-approved compounds using LRPPRC isogenic cancer cell models to identify agents that synergize with LRPPRC degrader-based OCBI therapy. The synergistic effects of the candidate compound were validated in multiple cancer cell lines with either genetic ablation or pharmacological inhibition of LRPPRC. Mechanistic studies were performed to investigate the impact on OXPHOS gene expression from both nuclear and mitochondrial genomes. **Results**: The clinically approved multi-kinase inhibitor Dasatinib was identified as a robust synergistic candidate, exhibiting heightened sensitivity in cancer cells with either LRPPRC knockout or pharmacological inhibition. Mechanistically, Dasatinib selectively suppressed nuclear-encoded OXPHOS genes, whereas LRPPRC inhibition preferentially impaired mitochondrial DNA-encoded OXPHOS genes, resulting in a coordinated dual-genome blockade of OXPHOS. **Conclusions**: This study uncovers a previously unrecognized synergistic anti-tumor effect between LRPPRC inhibition and Dasatinib, mediated by complementary suppression of nuclear- and mitochondrial genome-encoded OXPHOS pathways. These findings provide a strong mechanistic and translational rationale for combination therapies targeting LRPPRC-high tumors.

## 1. Introduction

Mitochondrial oxidative phosphorylation (OXPHOS) is a critical determinant of the occurrence and development of tumors by supplying both energy and biosynthetic precursor molecules essential to support tumor growth [1]. Notably, tumor subpopulations with high metastatic potential, including cancer stem cells (CSCs) and circulating tumor cells (CTCs), predominantly rely on OXPHOS rather than glycolysis to meet their bioenergetic demands [2,3]. Consequently, OXPHOS-targeting strategies represent a promising avenue receiving growing interest for cancer therapy. However, current OXPHOS inhibitors primarily disrupt energy production by suppressing the activity of a single OXPHOS complex. This strategy faces limitations such as inadequate blockade efficacy (inhibiting a single complex is insufficient to completely abrogate OXPHOS activity) and substantial side effects (all normal cells also rely on OXPHOS for energy production) [4]. These limitations underscore a critical unmet need for developing strategies that achieve more effective and tumor-specific disruption of OXPHOS.

Thirteen OXPHOS subunits encoded by mitochondrial genes are essential for assembling functional OXPHOS complexes [5]. The RNA-binding protein LRPPRC stabilizes these mitochondrial transcripts and coordinates mtDNA expression [6]. LRPPRC is frequently overexpressed in epithelial-derived tumors, where it acts as a pivotal factor augmenting OXPHOS metabolism [7,8,9,10,11,12]. This prompted our development of OXPHOS Complex Biogenesis Inhibition (OCBI), a strategy using small molecules (e.g., Gossypol Acetate, T96) to degrade LRPPRC [13]. OCBI selectively blocks newly synthesized OXPHOS complexes without affecting pre-existing complexes. As a result, normal tissues, which are characterized by low LRPPRC expression and slow mitochondrial turnover, are largely unaffected. Conversely, tumor cells are highly vulnerable to OCBI due to their heavy dependence on mitochondrial biogenesis, driven by rapid proliferation and remarkably active mitochondrial turnover (e.g., via autophagy). LRPPRC degraders based on OCBI show promising preclinical efficacy and are under clinical evaluation [14,15]. However, determinants of tumor sensitivity and optimal combination strategies remain unclear, hindering broader therapeutic application.

Our previous research has already demonstrated that both lung adenocarcinoma and triple-negative breast cancer rely on OXPHOS for energy production and are sensitive to GAA [9,10]. In this work, we conducted the first high-throughput screen to identify small-molecule compounds that act synergistically with LRPPRC degrader-based OCBI therapy, using cell models of lung adenocarcinoma (A549) and triple-negative breast cancer (MDA-MB-231). To identify synergistic agents, we assessed the differential sensitivity of isogenic cell pairs (with/without LRPPRC knockout) to a panel of FDA-approved compounds. After screening, the multi-target kinase inhibitor Dasatinib is identified as a potent synergistic partner of LRPPRC inhibition. Functional assays and transcriptomic profiling analysis reveal a complementary mechanism in which Dasatinib specifically suppresses nuclear-encoded OXPHOS genes, while LRPPRC inhibition preferentially impairs mtDNA-encoded OXPHOS expression. Collectively, this study uncovers a previously unrecognized metabolic vulnerability and provides a mechanistic rationale for combination therapies targeting LRPPRC-high tumors.

## 2. Results

### 2.1. LRPPRC Deficiency Inhibits Tumor Cell Proliferation

**Figure 1:**
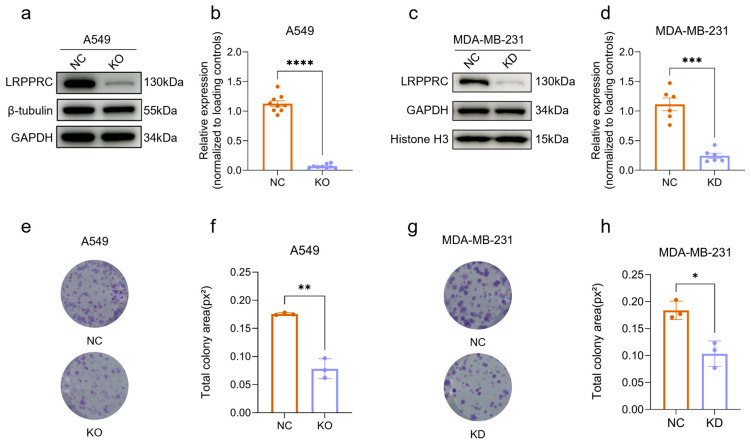
Effect of LRPPRC deficiency on tumor cell proliferation (**a**) Western Blot analysis of LRPPRC, β-tubulin, and GAPDH levels in LRPPRC knockout (KO) and NC A549 cells. (**b**) Quantification of LRPPRC protein level in (a, *n* = 9). Data are presented as mean ± SD, **** *p* < 0.0001. (**c**) Western Blot analysis of LRPPRC, GAPDH, and Histone 3 levels in LRPPRC knockdown (KD) and NC MDA-MB-231 cells. (**d**) Quantification of LRPPRC protein level in (c, *n* = 6). Data are presented as mean ± SD, *** *p* < 0.001. (**e**) Colony formation of NC and LRPPRC-KO A549 cells. (**f**) Quantification of colony area in (e, *n* = 3). Data are presented as mean ± SD, ** *p* < 0.01. (**g**) Colony formation of NC and LRPPRC-KD MDA-MB-231 cells. (**h**) Quantification of colony area in (g, *n* = 3). Data are presented as mean ± SD, * *p* < 0.05.

To investigate the effect of LRPPRC on cell proliferation, LRPPRC gene-knockout (KO) A549 cells were generated using the CRISPR/Cas9 system, and stable LRPPRC knockdown (KD) MDA-MB-231 cells were established through lentiviral shRNA transduction. The LRPPRC KO and KD efficiencies were validated by Western Blot assays showing a 93.9% reduction in A549 cells, and 78.2% reduction in MDA-MB-231 cells (Figure 1a–d). Colony formation assays showed that LRPPRC deficiency reduced the clonogenic growth in both A549 and MDA-MB-231 cells by 48% and 43%, respectively (Figure 1e–h). These results indicate that LRPPRC is a key regulator of tumor cell proliferation.

### 2.2. High-Throughput Screening Identifies Dasatinib as a Synergistic Anti-Tumor Compound with LRPPRC Inhibition

**Figure 2:**
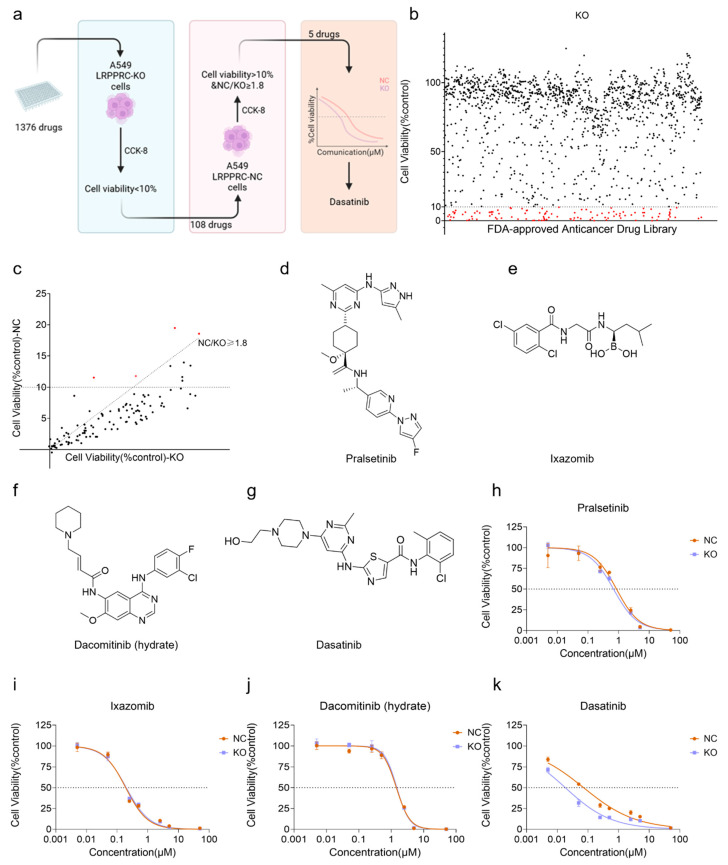
High-throughput screening identifies Dasatinib as a synergistic compound with LRPPRC inhibition. (**a**) Schematic diagram of the three-step screening strategy. Step 1: 1376 drugs were administered to A549 LRPPRC-KO cells at a single dose of 5 µM, and 108 drugs with a survival rate < 10% were selected; Step 2: These 108 drugs were tested on A549 LRPPRC-NC cells at 5 µM, and 4 candidates with NC cell viability >10% and NC/KO ratio ≥1.8 were retained; Step 3: Dose–response curves of the 4 candidates were determined in NC and LRPPRC-KO A549 cells at 8 concentration gradients, and Dasatinib was identified as the synergistic compound. (**b**) Viability of A549 LRPPRC-KO cells treated with 5 µM drugs in the primary screening, with 108 drugs selected (shown as red dots). (**c**) 4 candidates (shown as red dots) with a survival rate > 10% in NC cells and an NC/KO ratio ≥ 1.8 in the secondary screening. (**d**–**g**) Chemical structures of pralsetinib (**d**), lxazomib (**e**), dacomitinib (**f**), and Dasatinib (**g**). (**h**–**k**) Dose–response curves of four candidate compounds in NC and LRPPRC-KO A549 cells.

To identify compounds that exert a synergistic anti-tumor effect with LRPPRC inhibition, a “three-step” high-throughput screening was performed on 1376 FDA-approved anti-cancer drugs (Figure 2a). In the primary screening, LRPPRC-KO A549 cells were treated with a single dose of 5 µM of each drug, and 108 drugs with a survival rate < 10% were selected (Figure 2b; see Supplementary Table S1 for the complete list). For the 108 drugs, the most prominent categories include protein kinase inhibitors (e.g., Dasatinib, Cobimetinib, Trametinib, Crizotinib, and multiple ALK/EGFR inhibitors), anti-mitotic/microtubule-targeting agents, HDAC inhibitors, and natural products with multi-target activities. Subsequently, these 108 drugs were evaluated in A549-NC cells at 5 µM. 4 candidates preferentially inhibited the growth of LRPPRC-deficient A549 cells compared to NC cells, exhibiting greater than 1.8-fold change in cell viability. (Figure 2c–g). Finally, dose–response curves of these 4 drugs in NC and LRPPRC-KO A549 cells were determined at 8 concentration gradients (Figure 2h–k). Among them, Dasatinib, whose dose–response curve showed a significant leftward shift in LRPPRC-KO cells, was identified. Collectively, this rigorous screening strategy successfully identified Dasatinib as a compound that exhibits synergistic anti-tumor effects with LRPPRC deficiency.

### 2.3. Dasatinib Exhibits Synergistic Anti-Tumor Effects with LRPPRC Deficiency

**Figure 3:**
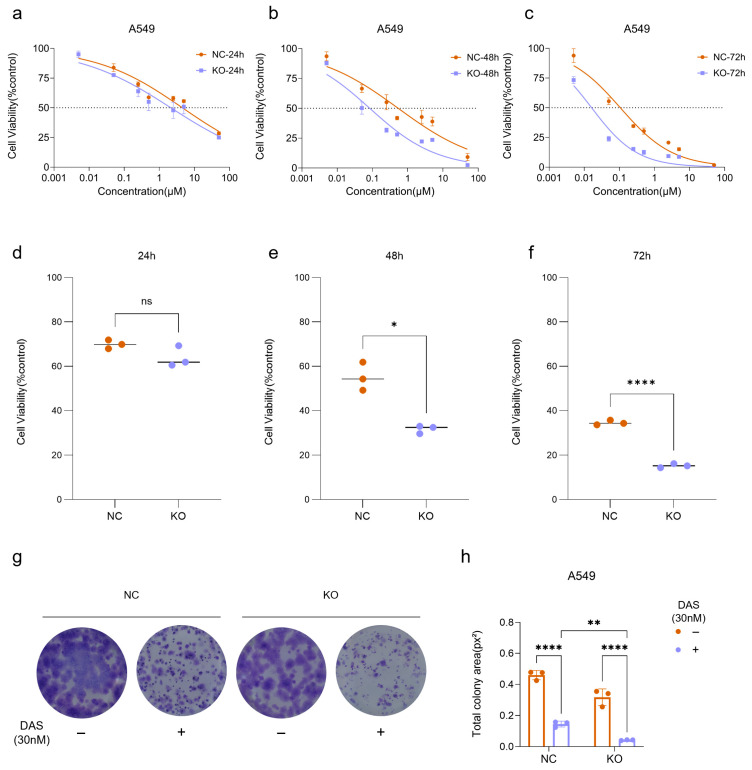
Dasatinib exhibits a synergistic anti-tumor effect with LRPPRC deficiency (**a**–**c**). Dose–response curves of A549 LRPPRC-KO vs. NC cells treated with Dasatinib for 24, 48, 72 h. (**d**–**f**) Cell viability of NC and LRPPRC-KO A549 cells treated with 0.25 µM Dasatinib for 24, 48, 72 h. Data are presented as mean ± SD, *n* = 3, ns indicates no statistical difference, * *p* < 0.05, **** *p* < 0.0001. (**g**) Colony formation of NC and LRPPRC-KO A549 cells treated with DMSO or 30 nM Dasatinib. (**h**) Quantification of colony area in (**g**). Data are presented as mean ± SD, *n* = 3, ** *p* < 0.01, **** *p* < 0.0001.

Dose-response curves revealed a time-dependent enhancement of Dasatinib sensitivity in LRPPRC-deficient cells (Figure 3a–f). From 24 to 72 h, the difference in cell viability between LRPPRC KO and NC cells progressively increased and became more significant at 0.25 μM Dasatinib (Figure 3d–f). The IC50 ratio between Dasatinib-treated NC and LRPPRC-KO A549 cells exceeded 6-fold (Table 1). Consistently, LRPPRC knockdown also enhanced the sensitivity of MDA-MB-231 cells to Dasatinib (Supplementary Figure S1a).

In addition, clonogenic assays further confirmed the synergistic effect between LRPPRC deficiency and Dasatinib treatment. Dasatinib (30 nM) treatment significantly reduced the colony-forming area of LRPPRC KO cells compared to NC cells (Figure 3g,h). A similar inhibitory effect was also observed in MDA-MB-231 cells (Supplementary Figure S1b,c). Collectively, these results demonstrate that genetic depletion of LRPPRC robustly enhances the anti-tumor efficacy of Dasatinib, supporting a synergistic anti-tumor effect between LRPPRC inhibition and Dasatinib.

### 2.4. The LRPPRC-Specific Degrader Gossypol Acetic Acid (GAA) Synergizes with Dasatinib to Suppress Tumor Cell Proliferation

**Figure 4:**
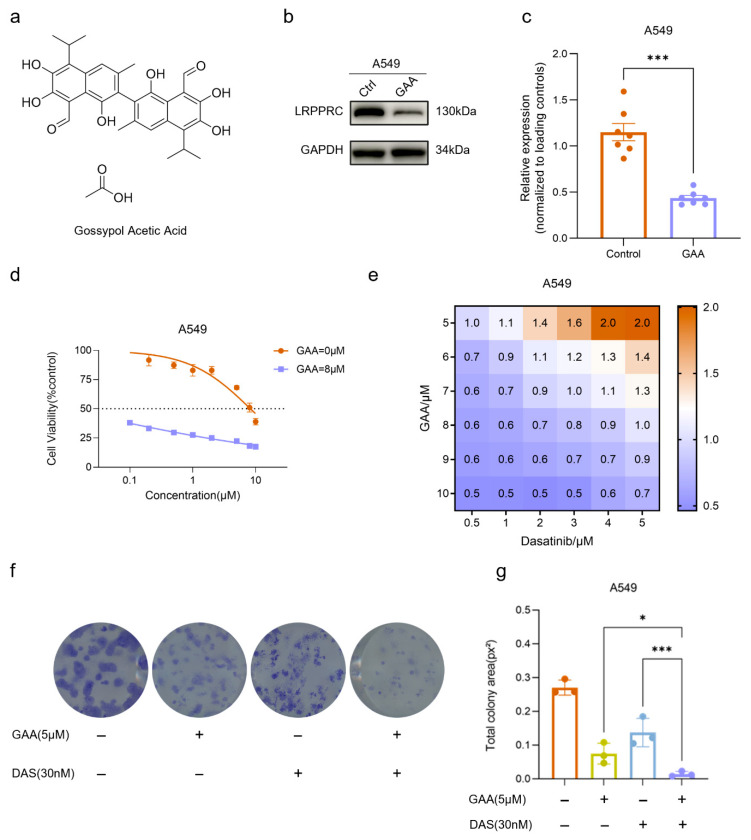
Synergistic anti-tumor effect of the LRPPRC degrader gossypol acetic acid (GAA) and Dasatinib (**a**). Chemical structure of GAA. (**b**) Western Blot analysis of LRPPRC protein level in A549 cells after treatment with 8 µM GAA for 24 h. Data are presented as mean ± SD, *n* = 7. (**c**) Quantification of LRPPRC protein level in (**b**). Data are presented as mean ± SD, *n* = 7, *** *p* < 0.001. (**d**) Sensitization effect of GAA on Dasatinib cytotoxicity. A549 cells were treated with Dasatinib alone (gradient concentrations) or Dasatinib (same gradient) combined with 8 µM GAA. (**e**) Combination index (CI) analysis of GAA (5, 6, 7, 8, 9, 10 µM) and Dasatinib (0.5, 1, 2, 3, 4, 5 µM), CI < 1 suggesting a synergistic effect. (**f**) Colony formation of A549 cells treated with GAA (5 µM), Dasatinib (30 nM), or GAA plus Dasatinib. (**g**) Quantification of colony area in (**f**). Data are presented as mean ± SD, *n* = 3. * *p* < 0.05; *** *p* < 0.001.

To further validate the synergistic anti-tumor activity between LRPPRC inhibition and Dasatinib, LRPPRC-specific degrader gossypol acetic acid (GAA, Figure 4a) was evaluated. GAA treatment alone inhibited the viability of A549 and MDA-MB-231 cells in a concentration-dependent manner, with IC50 values in the single-digit micromolar range, which is consistent with our previous report (Figure S2a,b), respectively [10]. Treatment with 8 µM GAA for 24 h dramatically reduced LRPPRC protein levels by 62.6% (Figure 4b,c). Consistently, 8 µM GAA treatment for 24 h also reduced LRPPRC protein levels by more than 60% in MDA-MB-231 cells (Supplementary Figure S2a,b). Next, the anti-tumor activities of GAA, Dasatinib, and their combination were tested in both cell lines. Co-treatment with GAA and Dasatinib significantly reduced cell viability compared to Dasatinib alone (Figure 4d). Furthermore, a 6 × 6 dose matrix of GAA (5, 6, 7, 8, 9, 10 µM) and Dasatinib (0.5, 1, 2, 3, 4, 5 µM) was analyzed to calculate the Combination Index (CI) via CompuSyn. Combination index analysis revealed multiple dose combinations with CI values < 1, indicating a synergistic anti-tumor effect (Figure 4e). A similar synergistic pattern was confirmed in MDA-MB-231 cells (Supplementary Figure S2c,d).

Consistent with these findings, colony formation assays demonstrated that combined GAA and Dasatinib treatment more effectively suppressed clonogenic growth than Dasatinib alone in both A549 (Figure 4f,g) and MDA-MB-231 cells (Supplementary Figure S2e,f). Collectively, these results demonstrate that pharmacological inhibition of LRPPRC phenocopies LRPPRC genetic depletion and synergizes with Dasatinib to inhibit tumor cell growth.

### 2.5. Dual-Genome Disruption of OXPHOS Genes Drives Synergy Between GAA and Dasatinib

**Figure 5:**
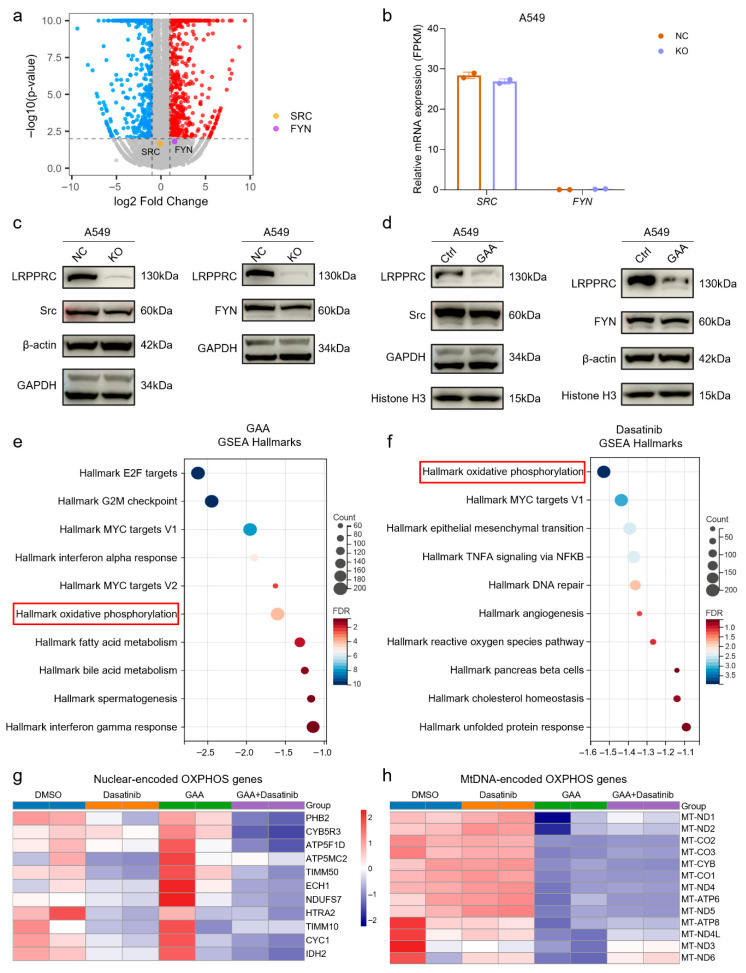
Dual-genome disruption of OXPHOS genes drives synergy between LRPPRC inhibition and Dasatinib (**a**) Volcano plot of gene expression in NC and LRPPRC-KO A549 cells; SFK members are colored. (**b**) The expression levels of *SRC* and *FYN* in NC and LRPPRC-KO A549 cells. (**c**) Western Blot analysis of Src and FYN protein levels in NC and LRPPRC-KO A549 cells. (**d**) Western Blot analysis of Src and FYN protein levels in A549 cells treated with or without GAA. (**e**) Hallmark pathway analysis of GAA downregulated genes. (**f**) Hallmark pathway analysis of Dasatinib downregulated genes. (**g**) Analysis of nuclear-encoded OXPHOS genes following GAA, Dasatinib, and their combination treatment. (**h**) Analysis of mitochondrial-encoded OXPHOS genes following GAA, Dasatinib, and their combination treatment.

To investigate the molecular mechanism underlying the synergistic effect between LRPPRC inhibition and Dasatinib, we performed transcriptomic profiling of NC and LRPPRC-KO A549 cells. Given that Dasatinib is an inhibitor of Src family kinases (SFKs) [16], we first examined whether LRPPRC deficiency altered the expression of SFK family members. Transcriptional analysis revealed that two primary SFK targets of Dasatinib, *SRC* and *FYN*, showed no significant change in mRNA levels following LRPPRC knockout (Figure 5a,b). These results indicate that the enhanced sensitivity to Dasatinib observed in LRPPRC-deficient cells is not mediated by transcriptional regulation of its classic SFK target. Consistent with these results, Western Blot analysis further confirmed that protein levels of Src and FYN did not change significantly in A549 and MDA-MB-231 cells following either genetic knockout or pharmacological inhibition of LRPPRC (Figure 5c,d, and Supplementary Figure S3a–d).

To further elucidate the mechanism underlying the synergistic effect between GAA and Dasatinib, we performed transcriptomic profiling of A549 cells treated with GAA, Dasatinib, or their combination. Hallmark pathway with gene set enrichment analysis revealed that only c-Myc target and oxidative phosphorylation pathways were suppressed by both GAA and Dasatinib (Figure 5e,f). Since OXPHOS complexes are encoded by both mitochondrial and nuclear genomes, and LRPPRC plays a key role in regulating mitochondrial OXPHOS gene expression [17,18], we hypothesized that their synergistic anti-tumor effect arises from coordinated disruption of OXPHOS gene regulation at both mitochondrial and nuclear genome levels. To test the hypothesis, we analyzed OXPHOS genes and revealed a genome-specific regulatory pattern. Dasatinib alone had little impact on mitochondrial DNA-encoded OXPHOS genes but markedly suppressed nuclear-encoded OXPHOS genes (Figure 5g). In contrast, GAA selectively downregulated mitochondrial DNA-encoded OXPHOS genes, with minimal effects on the nuclear-encoded counterparts, suggesting that combined GAA and Dasatinib treatment exhibits a more pronounced inhibition of OXPHOS (Figure 5h).

These findings revealed a previously unrecognized synergistic mechanism in which impaired LRPPRC function via GAA-mediated degradation preferentially suppresses mitochondrial genome-encoded OXPHOS components, while Dasatinib specifically inhibits nuclear-encoded OXPHOS genes. This mitochondria-nuclear coordinated disruption of both regulatory axes provides transcriptional evidence for the synergistic anti-tumor effects between LRPPRC inhibition and Dasatinib.

## 3. Discussion

The incomplete efficacy and systemic toxicity of OXPHOS-targeting degraders have limited their clinical translation. In this study, we develop a rational combination strategy that coordinately targets mitochondrial and nuclear determinants of OXPHOS. Through an unbiased high-throughput screen and mechanistic transcriptomic analysis, we demonstrate that the multi-kinase inhibitor Dasatinib synergizes with LRPPRC-specific degrader-mediated OCBI, resulting in enhanced anti-tumor activity.

LRPPRC has been reported to regulate the expression of mitochondrial DNA-encoded OXPHOS [17,19]. In line with these functions, genetic and pharmacological inhibition of LRPPRC in this study impaired tumor cell proliferation and clonogenic formation with selective downregulation of mitochondrial genome-encoded OXPHOS genes. These findings support LRPPRC as a therapeutic target. However, targeting mitochondrial respiration alone is often insufficient to fully suppress tumor growth, emphasizing the importance of developing combination strategies. Our screen strategy identified Dasatinib as a synergistic partner of LRPPRC inhibition. Although Dasatinib is known as an inhibitor of SFKs [16], neither transcriptomic nor protein analysis revealed significant changes in SFK gene expression following LRPPRC loss, suggesting that enhanced Dasatinib sensitivity is not mediated by altered SFK abundance. Instead, Hallmark pathway analysis revealed that Dasatinib unexpectedly disrupts nuclear-encoded OXPHOS genes, uncovering an additional insight into its anti-tumor activity.

The key mechanism from this study is the dual-genome regulation of OXPHOS by LRPPRC inhibition and Dasatinib. LRPPRC depletion preferentially suppressed mitochondrial DNA-encoded OXPHOS genes, while Dasatinib specifically inhibited nuclear-encoded OXPHOS genes. Combined treatment, therefore, results in a dual-genome disruption of OXPHOS. This dual-genome blockage provides a mechanistic explanation for the synergistic anti-tumor effects observed in different cell lines and highlights the mitochondrial-nuclear coordination as a critical determinant of metabolic vulnerability. Our study provides a strong rationale for combining LRPPRC-targeting strategies with Dasatinib or other drugs that impair nuclear-encoded mitochondrial programs. As Dasatinib and GAA are clinically approved, this combination is potentially extended to clinical evaluation, particularly in LRPPRC-high and OXPHOS-dependent tumors. In addition, the efficacy of this combination in mice needs to be assessed to determine whether similar dual-genome metabolic vulnerabilities can be leveraged with additional target therapies.

In conclusion, this study identifies a previously unrecognized synergy between LRPPRC inhibition and Dasatinib that is mediated by coordinated suppression of mitochondrial- and nuclear-encoded OXPHOS genes. These findings expand our understanding of how dual-genome regulation of mitochondrial respiration can be therapeutically exploited and provide a mechanistic framework for the development of combination strategies.

## 4. Method Details

### 4.1. Cell Culture

A549 and MDA-MB-231 cell lines were obtained from the American Type Culture Collection (ATCC, Manassas, VA, USA). A549 cells were cultured in RPMI 1640 medium (Gibco, Waltham, MA, USA) containing 10% fetal bovine serum (FBS, Gibco, USA) at 37 °C in a 5% CO2 incubator. MDA-MB-231 cells were cultured in DMEM medium (Gibco, Waltham, MA, USA) containing 10% fetal bovine serum (FBS, Gibco, USA) under the same conditions in a sterile environment. All cells were passaged regularly, and cells in the logarithmic growth phase were used for subsequent experiments. All cell lines were confirmed to be free of mycoplasma contamination by regular PCR testing.

### 4.2. In Vitro Chemical Screens

LRPPRC-KO A549 cells (3000/well) were seeded onto 96-well plates for 24 h. For the initial screen, cells were treated with a small molecule library (FAD-approved anticancer drug library) at a final concentration of 5 μM for 72 h. For the second screen, A549-NC cells (3000/well) were seeded onto 96-well plates for 24 h and then treated with candidate drugs screened in the first step at a final concentration of 5 μM for 72 h. For the third screen, LRPPRC-NC and KO A549 cells were treated for 72 h with 8 concentration gradients of chemicals (50 µM as the highest concentration) for IC50 determination. The cell viability was measured by the CCK-8 assay.

### 4.3. Combination Index (CI)

GAA and Dasatinib were individually diluted to generate concentration gradients and their corresponding combinatorial mixtures using complete medium. Cells were treated with single agents or the combination mixtures across a concentration gradient for 72 h. CI was calculated for each data point using the Chou-Talalay method with CompuSyn software (v1.2; ComboSyn, Inc., USA). Drug interactions were interpreted as follows: CI < 1, synergism; CI = 1, additive effect; CI > 1, antagonism.

### 4.4. Stable LRPPRC Knockdown Cells Generation

To generate a stable LRPPRC-knockdown cell line, lentiviruses encoding LRPPRC-specific shRNA sequences were obtained from Genechem Co., Ltd. (Shanghai, China). Cells were transduced with diluted lentiviruses for 48 h, then the medium was replaced with complete medium. Infected cells were selected using 2 μg/mL puromycin for an additional 48 h. shRNA sequence information: sh-LRPPRC: CCT CAA AGG AAT GCA AGA ATT.

### 4.5. Western Blot Analysis

Cells were lysed in RIPA lysis buffer (C1053-500, Applygen, Beijing, China) containing a protease inhibitor cocktail (K4002, APExBIO, Houston, TX, USA) and a phosphatase inhibitor (B15002, Selleckchem, Houston, TX, USA). Equal amounts of protein (10–40 µg) were separated by 10% SDS-PAGE and transferred to a PVDF membrane (IPVH00010, Merck Millipore, Burlington, VT, USA) for further analysis following standard Western blotting procedures. Primary antibodies were diluted with BSA at the following concentrations: Rabbit polyclonal anti-β-tubulin (10094-1-AP, 1:5000, Proteintech, Chicago, IL, USA), Rabbit polyclonal anti-GAPDH (10494-1-AP, 1:5000, Proteintech, Chicago, IL, USA), Rabbit polyclonal anti-β-actin (81115-1-RR, 1:5000, Proteintech, Chicago, IL, USA), Rabbit polyclonal anti-histone H3 (17168-1-AP, 1:5000, Proteintech, Chicago, IL, USA), Mouse monoclonal anti-LRPPRC (sc-390438, 1:2000, Santa Cruz Biotechnology, Dallas, TX, USA), Rabbit polyclonal anti-FYN (17237-1-AP, 1:1000, Proteintech, Chicago, IL, USA), Rabbit monoclonal anti-Src (clone 36D10; 2109, 1:1000, Cell Signaling Technology, Danvers, MA, USA). HRP-conjugated secondary antibodies (BS124446, 1:5000; BS124447, 1:5000, Bioworld, St. Louis, MO, USA) diluted in 5% BSA were incubated at room temperature for 2 h. Chemiluminescent signals were detected using an Amersham ImageQuant 800 imaging system (GE, Chicago, IL, USA), and the gray values of protein bands were quantified using ImageJ (v1.54f) software.

### 4.6. Colony Formation Assay

Cells were seeded in 12-well plates at a density of 300 cells per well. Twenty-four hours after seeding, drug treatment was performed simultaneously in the experimental group and the control group, and then the cells were continued to be cultured in a 37 °C incubator. Usually, after 10–14 days of culture, when colonies were observed to form, the cells were fixed with 4% paraformaldehyde for 20 min and stained with 0.1% crystal violet solution for 10 min. The stained colonies were imaged and analyzed using ImageJ software. Colonies consisting of >50 cells were counted. The colony formation rate was calculated and normalized to that of the control group. All experiments were independently repeated three times.

### 4.7. RNA-Seq Assay

A549 LRPPRC NC and KO cells were collected for analysis, while parental A549 cells were treated with DMSO, 5 μM GAA, 40 nM Dasatinib, or their combination for 24 h prior to collection. Total RNA was extracted from all samples using Trizol reagent. Samples were submitted to Astrocyte Technology (Hangzhou, China) for strand-specific library construction and sequencing on the Illumina NovaSeq X Plus platform with a PE150 strategy. Raw sequencing reads were subjected to quality control and filtering using Fastp software (v0.24.0) to remove adapter sequences and low-quality reads, generating high-quality clean reads. Subsequently, clean reads were aligned to the human reference genome GRCh38 (hg38) using HISAT2 (v2.2.1), and gene expression levels were quantified as FPKM values via StringTie (v1.3.4).

### 4.8. Statistical Analysis

All statistical analyses in this study were performed using GraphPad Prism software (v10.5.0). The number of biological replicates (*n* value) for each experiment is clearly indicated in the corresponding figure legends. Depending on the experimental design and data distribution characteristics, appropriate statistical methods were applied for between-group comparisons: Student’s *t*-test for comparisons between two independent samples, one-way analysis of variance (one-way ANOVA) for comparisons among multiple groups with a single factor, and two-way analysis of variance (two-way ANOVA) for comparisons among multiple groups with multiple factors. The threshold for statistical significance was defined as follows: ns, not significant; * *p* < 0.05, ** *p* < 0.01, *** *p* < 0.001, **** *p* < 0.0001.

Statistical analysis and visualization of transcriptomic sequencing data were performed using R software (v4.5.1). For A549 cells before and after LRPPRC knockout, a volcano plot drawn using the ggplot2 package (v4.0.0) was generated to display the distribution of differentially expressed genes. Genes with adjusted *p* value (adj. P) < 0.01 were considered significantly differentially expressed. For sequencing data from A549 cells treated with GAA, Dasatinib, or their combination, gene set enrichment analysis (GSEA) was performed on significantly down-regulated gene sets using the clusterProfiler package (v4.16.0) combined with Hallmark gene sets from the Molecular Signatures Database (MSigDB). Statistical significance of the enrichment analysis was adjusted for multiple testing using the Benjamini–Hochberg method, with an adjusted adj. *p* < 0.05 set as the threshold for significant enrichment to ensure result reliability. The GSEA results were visualized using bubble plots (generated with the ggplot2 package, v4.0.0) and heatmaps (generated using the pheatmap package, v1.0.13).

## 5. Conclusions

In conclusion, this study identifies a previously unrecognized synergy between LRPPRC inhibition and Dasatinib, which is mediated by the coordinated suppression of mitochondrial- and nuclear-encoded OXPHOS genes. These findings expand our understanding of how dual-genome regulation of mitochondrial respiration can be therapeutically exploited, and provide a mechanistic framework for developing combination strategies against LRPPRC-high and OXPHOS-dependent tumors. The clinical availability of both Dasatinib and LRPPRC degraders (e.g., GAA) further supports the translational potential of this combination therapy.

## Figures and Tables

**Table 1:** Inhibitory effect of Dasatinib on A549 LRPPRC non-knockout (NC) and knockout (KO) cells at different time points.

Time Point	Group	IC_50_ (μM)	95% CI (μM)	Fold Change (NC/KO)
24 h	NC	4.56	2.93–7.53	2.13
	KO	2.14	1.37–3.48	
48 h	NC	0.54	0.33–0.87	6.43
	KO	0.084	0.05–0.12	
72 h	NC	0.106	0.08–0.14	6.63
	KO	0.016	0.01–0.02	

Note: Data are presented to reflect measurement precision. The calculated fold-change in sensitivity (NC/KO) is shown.

## Data Availability

The original contributions presented in this study are included in the article/Supplementary Material. Further inquiries can be directed to the corresponding authors.

## Supplementary Materials

The following supporting information can be downloaded at: https://www.mdpi.com/article/10.3390/ph19030472/s1.
