# Supplementary material for: Synergistic Anti-Tumor Activity of LRPPRC Inhibition and Dasatinib Through Dual Oxidative Phosphorylation Disruption
Source: Pharmaceuticals (Basel). 2026 Mar 12;19(3):472. doi: 10.3390/ph19030472 (PMC13029704; doi:10.3390/ph19030472)
Supplement: Supplementary file 1 [file pharmaceuticals-19-00472-s001.zip › pharmaceuticals-4150050-supplementary.pdf]

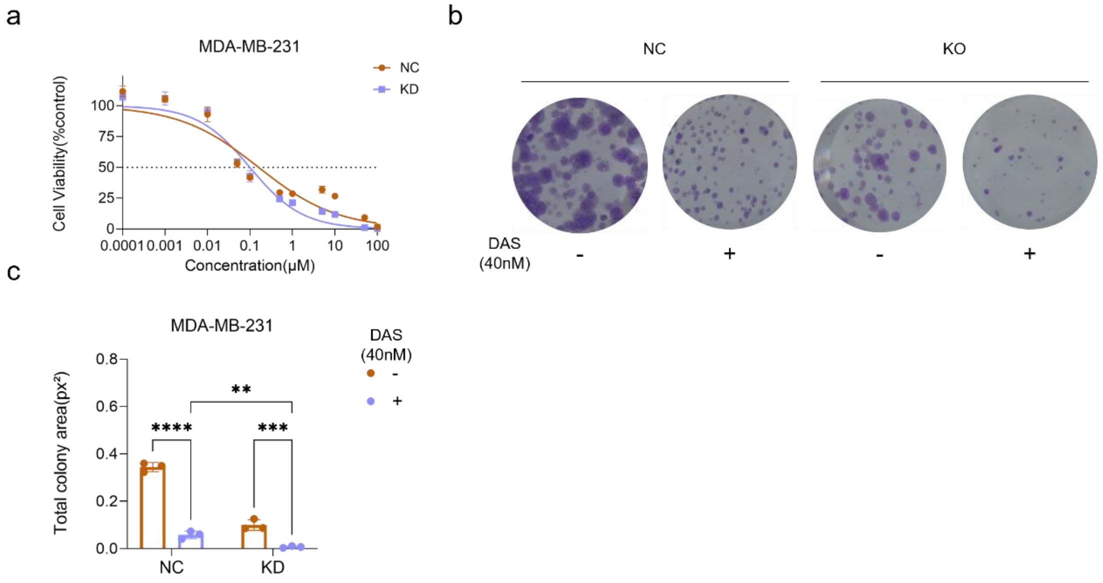

Supplementary Figure S1. Validation of the synergistic effect between LRPPRC deficiency and Dasatinib in MDA-MB-231 cells.

(a) Dose–response curves of MDA-MB-231 cells to Dasatinib in LRPPRC knockdown (KD) and negative control (NC) groups (72 h treatment). Data are presented as mean ± SD, n = 3.

(b) Colony formation of NC and LRPPRC-KD MDA-MB-231 cells treated with or without 40 nM Dasatinib.

(c) Quantification of colony area in (b, n=3). Data are presented as mean ± SD, **P < 0.01, ***P < 0.001, ****P < 0.0001.

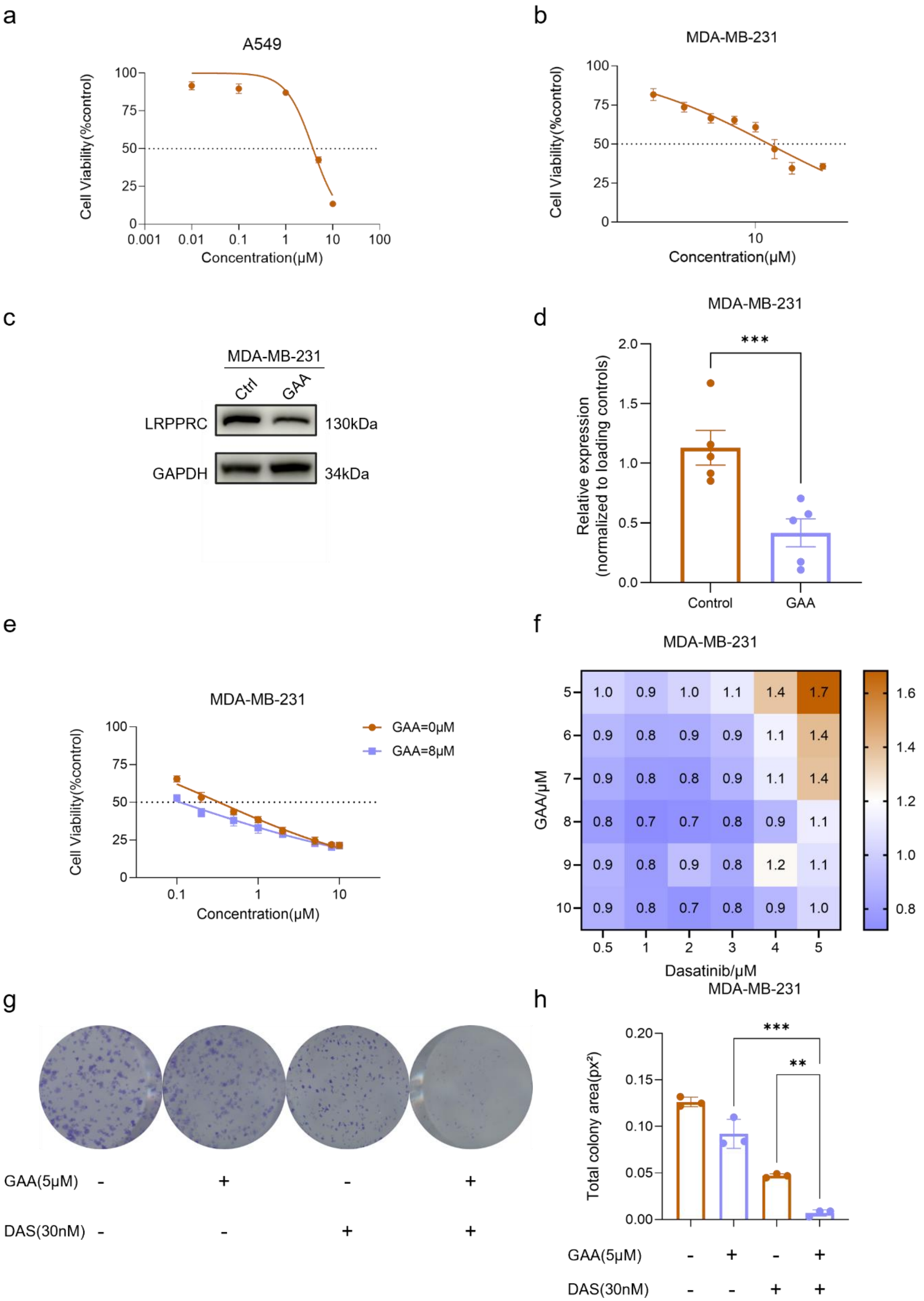
a
A549
Cell Viability(%control)
100
75
50
25
0
0.001
0.01
0.1
1
10
100
Concentration(μM)
b
MDA-MB-231
Cell Viability(%control)
100
75
50
25
0
10
Concentration(μM)
c
MDA-MB-231
Ctrl
GAA
LRPPRC
130kDa
GAPDH
34kDa
d
MDA-MB-231
***
Relative expression
(normalized to loading controls)
2.0
1.5
1.0
0.5
0.0
Control
GAA
e
MDA-MB-231
GAA=0μM
GAA=8μM
Cell Viability(%control)
100
75
50
25
0
0.1
1
10
Concentration(μM)
f
MDA-MB-231
GAA/μM
5 1.0 0.9 1.0 1.1 1.4 1.7
6 0.9 0.8 0.9 0.9 1.1 1.4
7 0.9 0.8 0.8 0.9 1.1 1.4
8 0.8 0.7 0.7 0.8 0.9 1.1
9 0.9 0.8 0.9 0.8 1.2 1.1
10 0.9 0.8 0.7 0.8 0.9 1.0
0.5 1 2 3 4 5
Dasatinib/μM
1.6
1.4
1.2
1.0
0.8
g
GAA(5μM) - + - +
DAS(30nM) - - + +
h
MDA-MB-231
***
**
Total colony area(px²)
0.20
0.15
0.10
0.05
0.00
GAA(5μM) - + - +
DAS(30nM) - - + +

Supplementary Figure S2. Synergistic anti-tumor effect of the LRPPRC degrader gossypol acetic acid (GAA) and Dasatinib in MDA-MB-231 cells.

(a) Dose–response curves of A549 cells to GAA (72 h treatment). Data are presented as mean ± SD, n = 3.

(b) Dose–response curves of MDA-MB-231 cells to GAA (72 h treatment). Data are presented as mean ± SD, n = 5.

(c) Western Blot analysis of LRPPRC expression in MDA-MB-231 cells after 24 h treatment with 8 μM GAA.

(d) Quantification of LRPPRC protein level in (a, n=5). Data are presented as mean ± SD, ***P<0.001.

(e) Cytotoxicity of Dasatinib alone or combined with 8 μM GAA in MDA-MB-231 cells.

(f) 6 × 6 concentration matrix for GAA (5, 6, 7, 8, 9, 10 μM) and Dasatinib (0.5, 1, 2, 3, 4, 5 μM) with combination index (CI) analysis; CI < 1 indicates synergy.

(g) Colony formation of MDA-MB-231 cells treated with 30 nM Dasatinib and/or 5 μM GAA. (–: no drug treatment; +: 5 μM GAA/ 30 nM Dasatinib treatment).

(h) Quantification of colony area in (e, n=3). Data are presented as mean ± SD, **P < 0.01, ***P < 0.001.

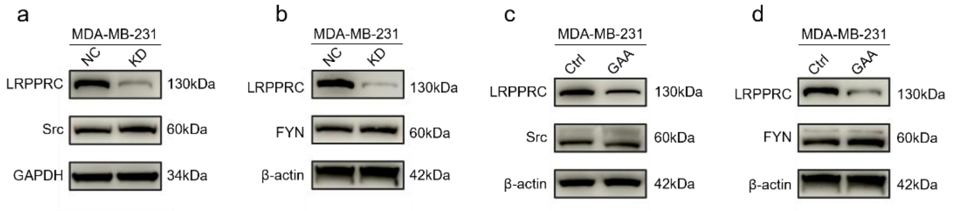

Supplementary Figure S3. Effect of LRPPRC knockdown/degradation on Src and FYN expression in MDA-MB-231 cells.

(a–b) Western Blot analysis of Src and FYN in LRPPRC-KD and NC MDA-MB-231 cells.

(c–d) Western Blot analysis of Src and FYN in MDA-MB-231 cells before and after 24 h treatment with 8 μM GAA.

Supplementary Table S1. Classification of the 108 compounds identified from the initial screening.

Class/Categorys	Drug
Protein Kinase Inhibitors	Dasatinib, Cobimetinib, Trametinib, Crizotinib, Ensartinib, Neratinib, Brigatinib, Ponatinib, Gilteritinib, Imatinib, Pacritinib, Ceritinib, Entrectinib, Dacomitinib, Midostaurin, ALK-IN-1, Taletrectinib, Olverembatinib, Repotrectinib, Pralsetinib, Fedratinib, Fostamatinib, Sulfatinib, Infigratinib, Volasertib, Dinaciclib, Copanlisib, Pimitespib
Anti-mitotic Agents	Vinblastine, Vincristine, Vindesine, Vinorelbine, Colchicine, Demecolcine, Podofilox, Maytansine, Tirbanibulin, Ixabepilone, Albendazole, Mebendazole
HDAC Inhibitors	Givinostat, Mocetinostat, Panobinostat, Belinostat, Vorinostat
Anthracyclines / Topoisomerase Inhibitors	Doxorubicin, Pirarubicin, Idarubicin, Epirubicin, Daunorubicin, Mitoxantrone, Valrubicin, Camptothecin, Topotecan, Belotecan
Natural Products / Multi-target Agents	Triptolide, Triptonide, Cinobufotalin, Homoharringtonine, Harringtonine, Plicamycin, Dioscin, Artemisinin, Daidzein
Proteasome Inhibitors	Bortezomib, Ixazomib
Antifolate Agents	Trimetrexate, Pralatrexate
Nucleoside Analogs	Gemcitabine, Gemcitabine elaidate, Fludarabine
Metabolic / Mitochondrial Modulators	Niclosamide, Niclosamide olamine, Dipyrithione, Verteporfin, Digitoxin
Estrogen Receptor Modulators	Tamoxifen, Lasofoxifene
Nuclear Export Inhibitors	Selinexor
Alkylating Agents	Ifosfamide
Antiparasitic Agents	Pentamidine, Albendazole, Mebendazole
GPCR / Ion Channel Modulators	Nebivolol, Amiodarone, Tamsulosin, Silodosin
Glucocorticoids	Mometasone
Others	Prussian blue insoluble, Bardoxolone methyl, Omaveloxolone
